# Immunohistochemistry as a screening tool for NTRK gene fusions: results of a first Belgian ring trial

**DOI:** 10.1007/s00428-020-02921-6

**Published:** 2020-09-11

**Authors:** Koen De Winne, Laure Sorber, Suzan Lambin, Vasiliki Siozopoulou, Gabriela Beniuga, Franceska Dedeurwaerdere, Nicky D’Haene, Lionel Habran, Louis Libbrecht, Jacques Van Huysse, Birgit Weynand, Katrin Wouters, Patrick Pauwels, Karen Zwaenepoel

**Affiliations:** 1 Laboratory of Pathological Anatomy, Antwerp University Hospital (UZA), 2650 Edegem, Belgium; 2 Center for Oncological Research Antwerp (CORE), University of Antwerp (UAntwerp), 2610 Wilrijk, Belgium; 3 Institute of Pathology and Genetics, 6280 Loverval, Belgium; 4 Department of Pathology, AZ Delta, 8800 Roeselare, Belgium; 5 Department of Pathology, Erasme Hospital, Université Libre de Bruxelles (ULB), Route de Lennik 808, 1070 Brussels, Belgium; 6 Anatomopathology Department, Centre Hospitalier Universitaire (CHU) de Liège, 4000 Liège, Belgium; 7 Department of Pathology, University Hospital Saint-Luc, 1200 Sint-Lambrechts-Woluwe, Belgium; 8 Department of Pathology, AZ Sint Jan, 8000 Brugge, Belgium; 9 Department of Pathology, University Hospital Leuven, 3000 Leuven, Belgium; 10 Department of Pathology, Jessa Hospital, 3500 Hasselt, Belgium; 11 Biobank UZA/UAntwerpen, Antwerp University Hospital (UZA), Edegem, Belgium

**Keywords:** Immunohistochemistry, NTRK fusions, Ring trial, Cancer screening, TRK inhibitor

## Abstract

A Belgian ring trial for pan-TRK immunohistochemistry (IHC) staining was organised to harmonise pan-TRK IHC staining protocols and interpretation. As a reference method, the VENTANA pan-TRK Assay (clone EPR17341) on the Benchmark Ultra platform was selected. Six samples were selected: 2 negative, 2 fusion positive and 2 samples with wild-type endogenous TRK expression. Each participating laboratory stained the slides using their routine pan-TRK IHC and reported their results. In addition, they were asked to return one TRK-stained slide from each case. The coordinating lab evaluated these slides, compared them with the reference method and scored them. Two clones were used during the ring trial: A7H6R (Cell Signaling) and EPR17341 (Abcam/Ventana). Seven protocols achieved a sufficient performance mark, and three labs were advised to further optimise the protocol. Interpretation of pan-TRK IHC proved to be challenging in cases with physiological TRK expression. In addition, depending on the *NTRK* fusion partner, the staining can vary strongly in both intensity and staining pattern. Labs using the Ventana ready-to-use system based on the EPR17341 clone and using the recommended protocol settings scored best. However, given some small optimisation, all labs scored well on the technical staining and the succeeding evaluation.

## Introduction

The neurotrophic tyrosine receptor kinases (NTRK, or commonly used TRK) are a family of transmembrane tyrosine kinases. TRKA, TRKB and TRKC proteins are encoded by the proto-oncogenes *NTRK1*, *NTRK2*, and *NTRK3* respectively and are physiologically expressed in the testes, smooth muscle and central and peripheral nervous system [1, 2]. Oncogenic fusions involving the kinase domain of the *NTRK* genes have been identified with high prevalence in certain rare cancers like infantile fibrosarcoma or secretory carcinoma of the breast [3]. The most common form of *NTRK* fusion gene, *ETV6-NTRK3*, is present in about 70% of infantile fibrosarcoma, making it a defining diagnostic feature [4]. More recently, *NTRK* fusions have also been identified in a small percentage of common cancers [5, 6], like soft tissue sarcomas [7], gliomas [8] and carcinomas of the lung [9], colon [10] and thyroid [11]. Farago et al. estimated the frequency of *NTRK* fusions in non-small-cell lung cancer (NSCLC) to be 0.23% [9]. A similar result was found in a cohort of 11,500 patients with various solid tumours, where only 0.27% harboured *NTRK* fusions [12]. Less than 0.31% of colorectal carcinomas are found to be *NTRK* fusion positive, but rates are substantially higher in the high microsatellite instability (MSI-H) phenotype [13]. Typically, the 5′ region of a partner gene fuses with the 3′ region of an *NTRK* gene, resulting in ligand-independent receptor activation [14].

TRK inhibitors have shown great promise as therapy for patients with tumours harbouring *NTRK* gene fusions. Clinical trials revealed high response rates, durable responses and favourable safety profiles. Furthermore, clinical responses were seen regardless of patient age, fusion partner, *NTRK* gene and tumour type [15, 16]. This has led to fast approval by the Food and Drug Administration (FDA) of TRK inhibitors for *NTRK* fusion positive solid tumours instead of for specific tumour types. First approved in 2019 in Japan, the multikinase inhibitor entrectinib exhibits activity against *NTRK*, *ROS1* and *ALK* oncogenic fusions. Larotrectinib, a highly selective TRK inhibitor received approval by the European Medicines Agency (EMA) in 2019.

As a consequence, identification of *NTRK* fusions has become vital for therapeutic management, and in some tumour types for diagnostic purposes. However, the presence of three different *NTRK* genes, combined with a high number of potential fusion partners and several possible breakpoints, makes the detection of *NTRK* fusions rather complex. A variety of techniques, like next-generation-sequencing (NGS), DNA and RNA-based assays or fluorescence in situ hybridisation (FISH) can be used to detect these fusions at the DNA, RNA or protein level [17]. In contrast to these assays, the use of immunohistochemistry (IHC) provides several benefits like a quick turnaround time, lower cost, wide availability and use of very limited tissue. Antibodies for IHC can be directed against specific TRK proteins [18] or can target an amino acid sequence common to TRKA, TRKB and TRKC (pan-TRK antibodies) [19]. Especially in low probability cancers (frequency < 5%), the use of a two-step approach is often suggested for *NTRK* fusion detection: The first step involves IHC as a screening or enrichment tool; the second step is to confirm the presence of a fusion by an RNA-based NGS analysis [20, 21].

Currently, there is limited experience with *NTRK* gene fusion testing in Belgium. The lack of an external quality assessment (EQA) for the pan-TRK IHC staining prompted us to organise a pan-TRK IHC ring trial. The key aim was to evaluate the reproducibility of TRK staining across different institutions using the same well-characterised samples and to provide feedback aimed at standardising the implementation and interpretation of TRK staining protocols. In addition, we also assessed the inter-observer variability in the evaluation of TRK IHC among pathologists.

## Materials and methods

### Sample selection

A total number of six formalin-fixed paraffin-embedded (FFPE) tissues were selected from the archives of the Antwerp University Hospital (UZA). The samples represented a mix of tumours, *NTRK* fusion positive and *NTRK* fusion negative samples. Besides tumour tissue harbouring a *NTRK* fusion, also tissue with endogenous TRK expression was included. The *NTRK* fusion status and possible fusion partner of the samples were confirmed by NGS testing. Targeted RNA-based NGS was conducted with the Oncomine Focus Assay (OFA) panel (Thermo Fisher Scientific, San Francisco, CA) on an S5 instrument, according to the manufacturer’s recommendations.

### Pan-TRK IHC

As a reference method we used the VENTANA pan-TRK assay (clone EPR17341) performed according to the instructions of the vendor on a Benchmark Ultra (Ventana Medical Systems, Tucson, AZ). This widely used EPR17341 clone is reactive with a conserved proprietary peptide sequence from the C-terminus of TRKA, TRKB and TRKC and is therefore reactive with any of the oncogenic TRK proteins. Tumours are considered positive if ≥ 1% of tumour cells exhibit staining at any intensity above background [12]. In addition, the different subcellular staining patterns (cytoplasmic, membranous, nuclear and peri-nuclear) are all considered to be positive. Staining intensity was quoted as negative (0), weak (1+), moderate (2+) or strong (3+).

### Design ring trial

The ring trial was coordinated by UZA and was conducted according to the Declaration of Helsinki, and ethical committee approval 18/49/577 was obtained on 07 January 2019 from the ethical committee of UZA. A total of 9 Belgian hospital labs participated in the ring trial. One laboratory participated with 2 protocols; therefore, the results will be discussed as if 10 labs participated.

The ring trial was setup according to the following steps:

1. The lead institute prepared blank slides from 6 resection cases. The first, middle and last slides were stained for pan-TRK to ensure similar staining patterns throughout the tissue.
2. Each participating laboratory received two blank coated slides to stain using their routine testing procedure. Labs were free to select their antibody and detection system of preference but were asked to provide information on the antibody, protocol and scoring method. Upon TRK staining, one or two pathologists of each centre reviewed and interpreted the staining. The laboratories were requested to return one TRK-stained slide per sample to the coordinating lab together with results of scoring and interpretation.
3. Once returned to the lead institute, the slide stained by the laboratories was compared with the reference staining and evaluated by a team of two experienced pathologists. Feedback was provided to the laboratories, including a technical evaluation and a comparison of the evaluation by the participating laboratories and by the team of trial-designated pathologists. Hereby, staining intensity, percentage of positive tumour cells and background staining were taken into account.

### Statistical analysis

To quantify the degree of agreement between the different observers, Cohen’s Kappa statistics (*K*) was used. It measures the inter-rater agreement for qualitative items and takes the possibility of the agreement occurring by chance into account. Kappa’s coefficient can range from − 1 to 1, where 0 represents the amount of agreement that can be expected from random chance. All statistical analyses were produced using SPSS statistics version 24 (IBM, Brussels, Belgium). The interpretation of Kappa’s coefficient is based on the Koch and Landis scale [22].

## Results

**Fig. 1:**
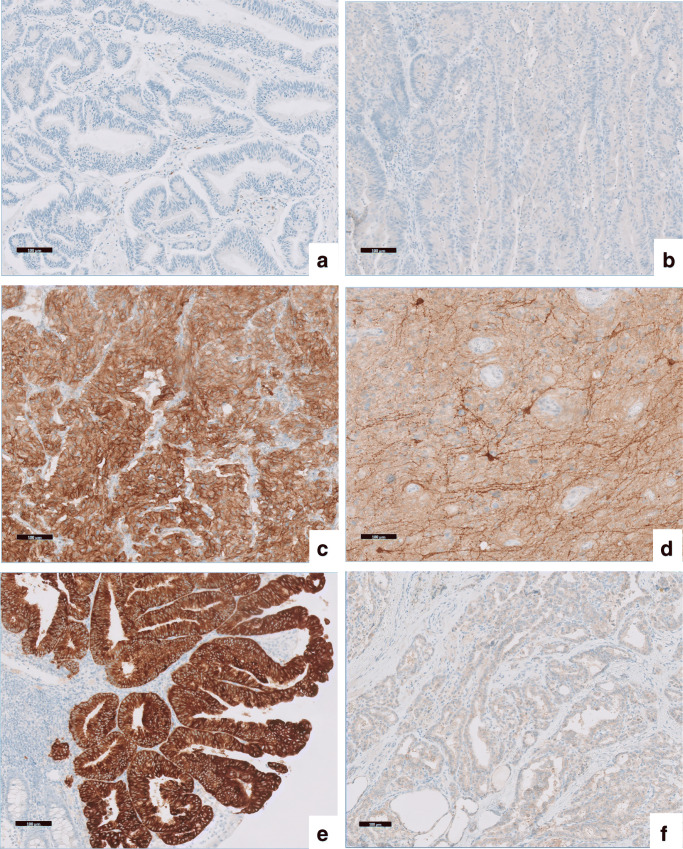
pan-TRK staining of ring trial samples. The scale bar indicates 100 μm. **a** Colorectal carcinoma. **b** Colorectal carcinoma. **c** Pheochromocytoma. **d** Glioma. **e** Colorectal MSI positive carcinoma. **f** Papillary thyroid carcinoma

**Fig. 2:**
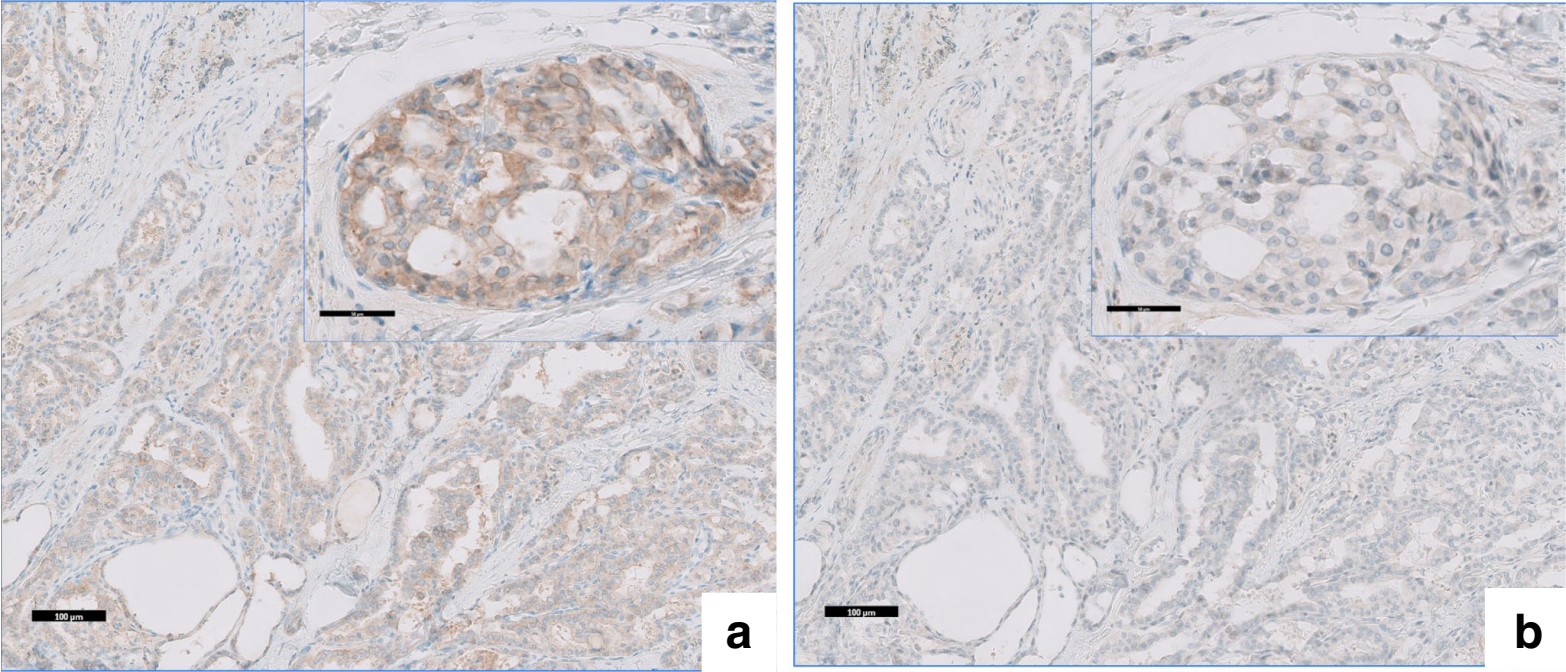
Papillary thyroid carcinoma. The scale bar represents 100 μm; the scale bar of the insert represents 50 μm. **a** Pan-TRK staining, **b** Negative control staining

The ring trial consisted of 6 samples: 2 negative samples, 2 samples with endogenous TRK expression and 2 samples with TRK fusion expressing tumour cells (Table 1). Stained with the VENTANA pan-TRK assay (clone EPR17341), the two negative samples did not show any expression (score 0; Fig. 1 a and b). The staining of the endogenous TRK expression of the pheochromocytoma case was predominantly granular cytoplasmic with membranous accentuation at variable intensities (score 1–3; Fig. 1c). Also, the glioma sample showed weak, diffuse staining of pre-existing brain tissue. The tumour cells however showed no staining (score 0; Fig. 1d). Both fusion-positive tumours demonstrated a cytoplasmic staining pattern: homogenous and strong staining in the microsatellite instability (MSI) positive colon carcinoma (score 3+; Fig. 1e) and diffuse and weak granular staining was present in the thyroid carcinoma (score 1+; Figs. 1f and 2).

**Table 1:** Overview samples

Sample	Cancer type	Result reference TRK staining
1	Colorectal adenocarcinoma	No TRK staining
2	Colorectal adenocarcinoma	No TRK staining
3	Pheochromocytoma	Endogenous TRK expression
4	Glioma	Endogenous TRK expression
5	Colorectal MSI positive adenocarcinoma	Strong cytoplasmic staining of 100% of tumour cells (*TPM3–NTRK1* fusion)
6	Papillary thyroid carcinoma	Weak to moderate cytoplasmic staining of 90% of tumour cell (*TPR–NTRK1* fusion)

Each of the participating laboratories stained the six slides with their routine testing procedure. After staining and analysing samples, they reported back to the coordinating lab. Each case was expected to be classified as either negative, positive or unclear. In addition, information was asked on staining percentage, intensity and clinical conclusion (fusion positive, fusion negative or further testing was required). The stained slides were afterwards sent back to the coordinating lab for evaluation by the designated trial pathologists.

### Technical evaluation

Pathologists from the coordinating lab evaluated the slides of the participating laboratories based on staining intensity, percentage of positive tumour cells and background staining (Table 2). These were then compared with the reference method and scored according to the following categories:

**Table 2:** Evaluation different protocols: I: intensity, %: percentage of positive tumour cells, B: background staining, 0: no staining, 1: weak staining, 2: moderate staining, 3: strong staining

Protocol	Samples																		Result
	1			2			3			4			5			6			
	I	%	B	I	%	B	I	%	B	I	%	B	I	%	B	I	%	B	
Reference	0	0	0	0	0	1	1–3	100	0	0	0	0	3	100	0	1–2	90	0	
1	0	0	0	0	0	1	1–3	100	0	0	0	0	3	100	0	2–3	100	1	Good
2	0	0	0	0	0	1	1–3	100	0	0	0	0	3	100	0	2	100	1	Good
3	0	0	0	0	0	1	1–3	100	0	0	0	0	3	100	0	1–2	100	0	Optimal
4	0	0	0	0	0	1	1–3	100	0	0	0	0	3	100	0	1–2	90	1	Good
5	0	0	0	0	0	1	1–3	100	0	0	0	0	3	100	0	0–1	70	0	Borderline
6	0	0	0	0	0	0	1–2	100	0	0	0	0	2–3	100	0	0	0	0	Poor
7	0	0	0	0	0	2	1–3	100	0	0	0	0	3	100	0	3	100	1	Good
8	0	0	0	0	0	1	1–3	90	0	0	0	0	3	100	0	0–1	80	1	Borderline
9	0	0	0	0	0	1	1–3	100	0	0	0	0	3	100	0	1–2	100	0	Optimal
10	0	0	0	0	0	1	1–3	100	0	0	0	0	3	100	0	1–2	90	0	Optimal

Optimal: The staining method scores identical to the reference method.

Good: The staining method shows an increased background staining, but diagnosis was correct.

Borderline: The staining method shows a decreased intensity of staining in tumour cells, but diagnosis was correct.

Poor: The staining method shows a decreased intensity of staining in tumour cells, generating a false negative result.

Compared with the pan-TRK assay reference method, seven protocols achieved a sufficient mark (optimal or good). Three labs were advised to further optimise their protocol (Table 3). Only two different antibody clones were used during the ring trial: A7H6R (Cell Signaling Technology) and EPR17341 (Abcam/Ventana). EPR17341 is available in a concentrated form (Abcam) or as a ready-to-use (RTU) assay by Ventana. Four labs (40%) used the RTU antibody, of which three followed the recommended in vitro diagnostic (IVD) settings and achieved the optimal mark (this protocol was also used as the reference method). The fourth lab added an amplification step, causing a slightly increased background staining (Table 3). The Ventana BenchMark was the most popular platform, being used by 80% of the labs. The remaining labs (20%) used the Dako Omnis platform. Both lab developed tests on Dako Omnis did not receive a sufficient mark, due to weak staining (Table 3). Seven participating laboratories (70%) used lab-developed tests. Because of increased background staining or decreased staining intensity, these lab-developed tests scored lower than the RTU assays (Tables 2 and 3).

**Table 3:** Assessment marks depending on antibody, platform and protocol: conc. AB: concentrated antibody, RTU AB: ready-to-use antibody, LDT: lab developed test, IVD: in vitro diagnostic test

		Percentage (number)	No. of optimal	No. of good	No. of borderline	No. of poor
Antibody	A7H6R	30 (3)	0	2	1	0
	EPR17341 conc. AB (Abcam)	30 (3)	0	1	1	1
	EPR17341 RTU AB (Ventana)	40 (4)	3	1	0	0
Platform	Ventana	80 (8)	3	4	1	0
	Dako Omnis	20 (2)	0	0	1	1
Protocol	LDT with conc. AB	60 (6)	0	3	2	1
	LDT with RTU AB	10 (1)	0	1	0	0
	IVD assay	30 (3)	3	0	0	0

### Evaluation of the analysis

To assess the inter-observer variability in the evaluation of pan-TRK IHC, the testing laboratory reported their results back to UZA and the stained slides of each lab were evaluated by two experienced pathologists. When comparing these results (being positive or negative), only one case gave a false positive discordant result, probably due to mild background staining (Fig. 1b). When interpreting the slides, nobody missed a positive case. Therefore, the false negative rate was 0%. To quantify the degree of agreement between the different observers, Cohen’s Kappa statistics (*K*) was used. In this case Kappa’s coefficient = 0.925 (*p* < 0.001), indicating an almost perfect degree of agreement between the different observers. However, besides positive or negative scores, the pathologist was also asked whether further testing was needed. When indicated this was necessary, the answer was always considered to be correct.

## Discussion

Oncogenic *NTRK* fusions are seen in many cancer types, but with the exception of some very rare tumour types, their incidence remains very low. These fusions have important therapeutic implications for patients with advanced cancers, making their routine detection a priority. Following the remarkable and often durable responses to TRK tyrosine kinase inhibitors, a wide range of techniques became available to detect the presence of *NTRK* fusions. Immunohistochemistry is a fast, cost-effective and widely available technique and provides an effective approach to screen for tumours harbouring *NTRK* fusions [23]. Especially in cases with a low probability of *NTRK* gene fusions, pan-TRK IHC can be performed as an enrichment strategy to select tumours for subsequent (RNA-based) NGS analysis. In contrast, for the rare subtypes that commonly harbour *NTRK* fusions (like infantile fibrosarcoma and secretory carcinomas of the breast and salivary glands) a histology-based triage followed by RNA-level fusion testing is suggested [23, 24]. The detection of RNA-level fusions provides direct evidence of functional transcription. In addition, splicing out of introns simplifies the technical requirements for detection of *NTRK* fusions, making RNA-based sequencing the preferred approach [20].

EPR17341 is a widely investigated pan-TRK clone and has demonstrated to be an efficient and reliable screening method for *NTRK* fusions [23]. Studies have shown sensitivities ranging from 75 to 92.5% and specificities between 81.1 and 100% [12, 19, 23, 25]. The A7H6R clone is also reactive with any of the TRK proteins but is less investigated than EPR17341. One study compared these two pan-TRK IHC clones in advanced melanoma tumour samples. Different staining results suggest that the clones do not target the same epitopes in the TRK proteins. In addition, the authors hypothesised that EPR17341 might be more specific—but perhaps also less sensitive—than A7H6R. Due to a lack of *NTRK*-rearranged tumours, no real comparison could be made [26]. In a recent study by Guibourg et al., a total of 71 salivary gland tumours were stained with the two pan-TRK IHC antibody clones. Only one case was fusion-positive, and this *NTRK3*-rearranged salivary secretory carcinoma was found to be positive with both the EPR17341 and the A7H6R clone [27]. Finally, in a large cohort of over 4000 colorectal cancer samples, both antibodies demonstrated similar staining characteristics and showed diffuse strong cytoplasmic staining in all nine fusion-positive cases. In addition, there was also complete concordance between the two observers in interpreting both antibodies [13].

Based on the results of this ring trial, the EPR17341 and A7H6R clones are both highly recommendable antibodies for pan-TRK IHC. Labs using the Ventana ready-to-use system based on the EPR17341 clone and following the recommended protocol settings scored best. However, given some small optimisation, labs using a lab developed test or labs using the A7H6R clone can also achieve a sufficient or optimal mark.

Interpretation of IHC TRK staining may seem straightforward, but can be more challenging than anticipated. The staining can vary strongly in both intensity and staining pattern, which often correlates with the subcellular localisation of the fusion partners. In contrast to the membrane-associated expression of native TRK, the fusion partner can direct the fusion protein to localise to other cell compartments. As a consequence, the staining pattern can be cytoplasmic, nuclear, perinuclear or membranous [19]. Currently, approximately 80 different 5′ *NTRK* gene fusion partners have been identified in a wide array of tumour types [14]. Also, the percentage of tumour cells with positive staining can vary among the tumours. This variation in staining pattern, intensity and percentage was illustrated by the two TRK positive samples included in the ring trial (Fig. 1 e and f). Both samples harbour an *NTRK1* fusion, resulting in a cytoplasmic staining pattern, but with very different intensity. In contrast to the strong, uniform staining of the MSI positive colon carcinoma, the papillary thyroid carcinoma showed only very weak diffuse staining. As a consequence, one of the sub-optimal staining protocols from the ring trial led to a false negative result. In these unclear borderline cases, including a negative control (IHC staining without addition of the primary antibody) can facilitate correct interpretation (Fig. 2 a and b).

Another potential pitfall in the interpretation of pan-TRK IHC is the occurrence of physiological TRK expression. Under normal circumstances wild-type TRK is expressed in smooth muscles, testes and neural components. Particularly challenging is the interpretation of glioma samples because of the positivity of normal brain tissue (Fig. 1d). In addition, also, a subset of tumours, like pheochromocytoma (a neural crest-derived tumour), express TRK, but without the presence of an *NTRK* gene rearrangement (Fig. 1c). The usefulness of TRK IHC in these two samples from the ring trial is limited, illustrating that selection of the appropriate assay for *NTRK* fusion detection also depends on tumour type. Especially in tumours with neural differentiation, specificity of pan-TRK IHC may be insufficient and RNA-based NGS analysis should be considered [23].

As reported in literature, an additional limitation for IHC analysis is the reduced sensitivity for *NTRK3* fusions [12, 23]. While sensitivities close to 100% (96% for *NTRK1* and 100% for *NTRK2*) were demonstrated, only 79% sensitivity was found for *NTRK3*. In some of these cases, staining was found to be weak and focal, increasing the chance of false negative results [23]. Therefore, it would be of great interest for a future EQA to include an *NTRK3* fusion positive sample.

There was an almost perfect degree of agreement (Kappa's coefficient 0.925, *p* < 0.001) in TRK IHC scoring between the pathologists of the participating laboratories and the trial-designated pathologists. Only one slide was interpreted differently by the observers. This is partly due to the—always considered correct—option to indicate extra testing was needed. Most labs scored the pheochromocytoma and glioma samples (Fig. 1 c and d) as positive, but also indicated the need for further testing. The available drugs (larotrectinib and entrectinib) are only useful in *NTRK*-rearranged tumours; no responses are seen in *NTRK* mutated or amplified tumours [5, 28]. Therefore, further testing to identify the cause of IHC staining is often needed. However, some of the labs even indicated the need for further testing, even in the absence of (tumoural) staining. In these labs, the efficiency of screening by IHC to detect TRK positive cases was limited.

The main purpose of this ring trial was to harmonise pan-TRK IHC staining protocols and subsequent interpretation. Despite the use of different antibodies and detection systems, TRK IHC staining results were comparable between the various laboratories, proving the robustness of these procedures. Care should be taken when endogenous expression occurs in the sample, in which case RNA-based NGS analysis is needed. To conclude, the use of IHC as a screening tool, followed by molecular testing to confirm the fusion partners, seems to be an effective approach, especially in case of tumour types with a low incidence of NTRK fusions. Finally, by including more diverse staining patterns and different *NTRK* fusion partners (especially *NTRK3* fusion positive samples), the educational value of future ring trials could increase.

## Data Availability

All data has been presented in the manuscript.
